# Early Diagnosis and Management of Congenital Afibrinogenemia: A Case Report of Umbilical Stump Bleeding

**DOI:** 10.7759/cureus.42542

**Published:** 2023-07-27

**Authors:** Qais M Salah, Rami R AlRashayda, Mohammad J Heresh, Aya Abulehya, Leena W Salah Al-deen

**Affiliations:** 1 Internal Medicine, Al-Quds University, Jerusalem, PSE; 2 Medicine, Al-Quds University, Jerusalem, PSE; 3 Pediatric Medicine, Palestine Medical Complex, Ramallah, PSE; 4 Pediatric Medicine, Specialized Arab Hospital, Nablus, PSE

**Keywords:** congenital fibrinogen disorder, bleeding disorder, congenital afibrinogenemia, coagulopathy, umbilical stump bleeding

## Abstract

Afibrinogenemia is an extremely rare bleeding disorder characterized by the absence or severe deficiency of fibrinogen, a major protein involved in the regulation of blood clotting. This disorder can have both hemorrhagic and/or thrombotic manifestations. We present the case of a female neonate who was diagnosed with congenital afibrinogenemia within the first two weeks of life. The patient presented with persistent bleeding from the umbilical stump, prompting a thorough investigation and workup. Early diagnosis and management were essential to preventing life-threatening bleeding events.

## Introduction

Fibrinogen, also known as coagulation factor I, plays a crucial role in hemostasis as it is converted to fibrin (factor Ia), a pivotal step in platelet aggregation and clot formation to regulate the process of wound healing. Congenital disorders of fibrinogen can be due to a partial deficiency in fibrinogen or, in some instances, a complete deficiency (afibrinogenemia). Other documented disorders involve dysfunctional fibrinogen production. Heritable disorders of fibrinogen are rare [1]. According to a global survey for rare bleeding disorders in 2014, inherited fibrinogen disorders accounted for 0.6% of the total bleeding disorders [2]. Acquired fibrinogen disorders are more common than inherited defects, in which liver disease is the most frequent etiology.

In neonates, afibrinogenemia is particularly concerning as it poses serious challenges due to its early onset and the potential for severe bleeding episodes. Bleeding due to fibrinogen disorders in the neonatal period may present at different sites, including mucous membranes, umbilical stumps, and bleeding following circumcision in male infants [3,4]. Therefore, it is crucial for healthcare professionals to recognize the signs and symptoms of afibrinogenemia in the neonatal period to initiate care and management rapidly.

In this case report, we present a four-day-old female neonate patient who presented with umbilical stump bleeding. The clinical presentation and diagnostic evaluation of the patient raised the suspicion of an inherited bleeding disorder.

## Case presentation

A full-term female neonate was born via spontaneous vaginal delivery to a 34-year-old multigravida mother. The antenatal period was unremarkable, with normal prenatal ultrasounds and laboratory investigations. The Apgar scores were 8 and 10 at one and five minutes, respectively. The infant's birth weight was 3.1 kg, and there were no complications after delivery. The patient and her mother were discharged on the second day of life after receiving the appropriate care and advice for the postpartum period.

On the fourth day of life, the mother noticed an oozing of blood from the umbilical stump, which continued despite applying pressure and packing the stump. No excessive trauma or manipulation had occurred. There was no history of similar episodes among the siblings, but family history was notable for two paternal aunts who died at the ages of three and four years, respectively, due to bleeding events. The infant appeared pale, had a heart rate of 150 beats per minute and blood pressure within the normal range for her age, and her overall activity was normal. Physical examination was remarkable for the constant oozing of blood from the umbilical stump, with no evidence of bleeding from mucosal surfaces or other sites.

Laboratory investigations were performed to assess the cause of the umbilical stump bleeding. The complete blood count results (Table 1) were within the normal range. Coagulation studies demonstrated a prolonged prothrombin time (PT) of 100 seconds (reference range: 10-12.5 seconds), prolonged activated partial thromboplastin time (aPTT) of 120 seconds (reference range: 27-40 seconds), and a significantly increased international normalized ratio (INR) of 8 (reference range: 0.9-1.3).

**Table 1:** Complete blood count for the patient upon presentation

Laboratory tests	Results	Reference range
Hemoglobin	17.5 g/dL	13-20 g/dL
Hematocrit	52%	46-62%
Red blood cell count	5.8×10^6^/μL	5-7 ×10^6^/μL
Platelet count	270×10^3^/μL	150-450 x10^3^/μL
White blood cell count	14x10^3^/μL	9-27 x10^3^/μL

Further screening for coagulation disorders was required. A mixing study with normal plasma was done to differentiate between factor deficiencies and factor inhibitors, and it was significant for improved PT, aPTT, and INR (Table 2). These findings suggested a coagulation factor deficiency, which warranted measuring factors’ activity and antigen levels. Testing revealed undetectable fibrinogen levels (reference range: 130-300 mg/dL), indicating the diagnosis of congenital afibrinogenemia. Moreover, genetic analysis revealed that the patient has homozygous mutations in the gene encoding for the fibrinogen α chain (FGA), which confirmed the diagnosis.

**Table 2:** Laboratory testing for the patient coagulation profile before and after the mixing study

Laboratory tests	Results before the mixing study	Results after the mixing study	Reference range
Prothrombin time (PT)	100 seconds	15 seconds	13-20 seconds
Activated partial thromboplastin time (aPTT)	120 seconds	29 seconds	27-40 seconds
International normalized ratio	8	1.27	0.9-1.3

The management of the infant’s condition included the administration of cryoprecipitate, which contains concentrated fibrinogen, to address the fibrinogen deficiency. Cryoprecipitate was administered at a dose of 1 unit per 5 kg of body weight, repeated as needed based on the patient's response. The infant's bleeding from the umbilical stump ceased within hours of cryoprecipitate infusion, and her laboratory tests normalized as well. Subsequently, the patient underwent regular monitoring of fibrinogen levels and received repeated administrations of cryoprecipitate units based on her fibrinogen levels.

## Discussion

Congenital afibrinogenemia is a rare disorder characterized by undetectable circulating fibrinogen levels, and has an estimated incidence of one case per million [5]. It usually occurs as an autosomal recessive condition in which patients are either homozygous or compound heterozygous for truncating mutations in the gene encoding for the fibrinogen α chain (FGA) [6]. These mutations affect fibrinogen production by different mechanisms, including mRNA splicing or stability, protein production or stability, or hexamer assembly, storage, or secretion [7].

Congenital afibrinogenemia is typically present in the neonatal period. An international cross-sectional study involving 204 patients with afibrinogenemia reported that one-third of patients had at least one bleeding event per month and one-fourth had a history of cerebral bleeding [8]. Other reported cases included umbilical cord bleeding, which can be a serious and fatal condition, and splenic rupture [9]. Some affected individuals have a later onset of symptoms, which can present as bleeding from the mucosal surfaces, the gastrointestinal tract, or intracranial bleeding. The majority of congenital fibrinogen disorders usually do not present with thrombotic manifestations. However, arterial and venous thrombotic complications have been reported [10].

The preliminary evaluation for suspected fibrinogen disorders includes a personal and family history of hemorrhagic, thrombotic, or obstetric complications. Measuring PT, aPTT, INR, and thrombin time (TT) is also recommended in suspected individuals. Mixing studies aid in the diagnosis as well, as they show correction and improvement of PT, aPTT, INR, and TT following the mixing of the patient's plasma with normal plasma. The diagnosis is typically confirmed through genetic sequence analysis of the fibrinogen genes.

The key goal in managing patients with fibrinogen disorders is to prevent and treat serious bleeding and any thrombotic or even obstetric complications. Routine prophylaxis for patients with fibrinogen disorders is usually not necessary. However, patients with afibrinogenemia or severe hypofibrinogenemia (e.g., fibrinogen <10 mg/dL) can be treated prophylactically with fibrinogen concentrates or cryoprecipitate. Furthermore, patients with a known history of previous bleeding events and those with congenital afibrinogenemia should receive fibrinogen replacement prior to surgery or elective procedures that carry a risk of bleeding [11]. In acute bleeding events, it is suggested to raise the functional fibrinogen level to >100 to 150 mg/dL until hemostasis is achieved. A target of 150 to 200 mg/dL can be utilized for patients with very severe bleeding (such as intracranial bleeding) [11].

Products for replacing fibrinogen include fibrinogen concentrates and plasma products. These products have not been compared to one another in randomized trials, and each one has its own advantages and disadvantages [12]. However, it is recommended to use fibrinogen concentrate, if available, rather than other sources of fibrinogen because it carries a lower risk of transfusion reactions and volume overload. Subsequent doses of fibrinogen replacement therapy are based on the patient’s trough plasma levels rather than a fixed dose or schedule, as pharmacokinetics vary widely among individuals [13].

Guidelines for the treatment of thrombosis in patients with fibrinogen disorders are limited. In general, thrombotic events should be treated with anticoagulants unless there is a contraindication. Low-molecular-weight heparins are the anticoagulants of choice for such events [14], but there is no data on the efficacy and safety of direct oral anticoagulants in these events. All patients with thrombophilic fibrinogen variants should be counseled about additional risk factors for thrombosis, signs and symptoms, and risk reduction strategies.

## Conclusions

Congenital afibrinogenemia is an infrequent condition, but it is life-threatening. Prompt diagnosis and management are crucial to improving a patient's outcomes. Our case emphasizes the importance of early recognition of this condition, as it can manifest within the first few days of life. Genetic testing remains the gold standard for diagnosis by detecting mutations affecting fibrinogen genes.
